# Response to Treatment with Melatonin and Clonazepam versus Placebo in Patients with Burning Mouth Syndrome

**DOI:** 10.3390/jcm11092516

**Published:** 2022-04-29

**Authors:** Candela Castillo-Felipe, Asta Tvarijonaviciute, Marina López-Arjona, Luis Pardo-Marin, Eduardo Pons-Fuster, Pia López-Jornet

**Affiliations:** 1 Colaborate Department Stomatology School of Medicine, Oral Medicine University of Murcia, 30100 Murcia, Spain; candela.casti@gmail.com; 2 Interdisciplinary Laboratory of Clinical Analysis, Interlab-UMU, Regional Campus of International Excellence ‘Campus Mare Nostrum’, University of Murcia, 30100 Murcia, Spain; asta@um.es (A.T.); marina.lopez10@um.es (M.L.-A.); lpm1@um.es (L.P.-M.); 3 Faculty of Medicine, Department Anatomy Psicobiology Regional Campus of International Excellence ‘Campus Mare Nostrum’, University of Murcia, 30100 Murcia, Spain; eduardo.p.f@um.es; 4 Department of Oral Medicine, Faculty of Medicine, Regional Campus of International Excellence ‘Campus Mare Nostrum’, IMIB Instituto Murciano de Investigación Biosanitaria-Arrixaca University of Murcia, 30100 Murcia, Spain

**Keywords:** burning mouth syndrome, pain, xerostomia, sleep, melatonin, clonazepam, placebo

## Abstract

Objective: to evaluate the efficacy of melatonin and clonazepam versus placebo in patients with burning mouth syndrome (BMS). Methods: a prospective double-blind study was carried out in patients with BMS and randomized to three groups: melatonin (1 mg once a day), clonazepam (0.5 mg/twice a day), or a placebo once a day, for 8 weeks. The clinical changes were evaluated, including xerostomia, the Oral Health Impact Profile 14 (OHIP-14) score, Pittsburg Sleep Quality Index, and the Hospital Anxiety and Depression Scale (HADS). Oxygen saturation and heart rate were recorded, with an analysis of salivary biomarkers in the forms of oxytocin, ferritin, adenosine deaminase (ADA), total proteins, and alpha-amylase. Results: a total of 64 patients were analyzed. A significant decrease in burning sensation was recorded with melatonin (7.8 ± 1.54 pre-treatment, 5.78 ± 2.54 post-treatment; *p* < 0.001) and clonazepam (8.75 ± 1.2 pre-treatment, 5.5 ± 3.6 post-treatment (*p* < 0.01). With regard to quality of life (OHIP-14), significant improvements were observed before and after the administration of melatonin (*p* < 0.001) and clonazepam (*p* = 0.001). On the other hand, with regard to the changes in salivary biomarkers following treatment, negative correlations were found between oxytocin and drainage (r = −0.410; *p* = 0.009) and between the HADS-D score and ferritin (r = −0.312; *p* = 0.05). While salivary amylase showed positive correlation with heart rate (r = 0.346; *p* = 0.029) and oxygen saturation (r = 0.419; *p* = 0.007). Conclusions: melatonin and clonazepam were shown to be effective at reducing the burning sensation and improving quality of life. Both drugs were found to be safe, with no major adverse effects in patients with BMS. Melatonin may be regarded as an alternative treatment for patients with BMS, though further studies are needed to confirm its effectiveness.

## 1. Introduction

Burning mouth syndrome (BMS) is characterized by chronic pain of the oral cavity, and is estimated to affect 0.1–3.9% of the population [1,2,3]. It is defined as a recurrent daily oral burning sensation or dysesthesia lasting over two hours a day for a period of over three months, in the absence of clinical lesions, capable of explaining the symptoms [4]. The precise etiology of BMS remains unclear [5,6,7]. This complicates patient management, and no definitive treatment for the disorder has been established to date [8]. Many studies have evidenced that in addition to neuropathic alterations, psychological factors are implicated in this disease [9,10,11]. In this regard, anxiety and depression are the most common psychopathological disorders observed in these patients [10]. Likewise, BMS seems to be related to endocrine and metabolic changes that can give rise to alterations of the hypothalamic–pituitary–adrenal axis, with an impact on salivary composition [12]. Adequate management of the pain requires due evaluation of its presence and severity [13,14]. In this regard, patient descriptions of the pain experience remain the gold standard for the evaluation of pain, and this is conditioned to adequate communication capacity. It is therefore of interest to establish biomarkers capable of contributing to the assessment of different aspects of BMS [14,15,16,17]. Saliva can offer noninvasive fluid samples of great diagnostic potential that may contribute to identify and monitor the disease. In this respect, the salivary alpha-amylase concentrations increase in situations of stress and activation of the sympathetic nervous system. On the other hand, adenosine deaminase (ADA) participates in different processes related to the immune system. In turn, oxytocin is a neuropeptide that plays a key role in health and has been implicated in the attenuation of pain [17,18,19,20,21,22,23,24]. It is important to note that increased salivary ferritin can change the properties of saliva and also has the potential to bring about changes in the oral environment. Ferritin, being acidic, can reduce the salivary pH and the buffering capacity of saliva; this can in turn lead to an increased incidence of dental caries [25].

Despite the advances in the knowledge and treatment of BMS, the disorder remains a challenge [8]. Very diverse therapeutic strategies have been evaluated, with controversial results [26,27,28,29] In this regard, the treatments used have included antiseizure drugs, benzodiazepines, antidepressants, analgesics, laser therapy, topical agents, physical barriers such as tongue protectors, psychotherapy, and nutritional supplements [8,25,26,27,28]. The best therapeutic outcomes have been recorded with clonazepam, with a significant decrease in pain following administration of the drug, though not all patients show a good response to this treatment. In 2016, Arduino et al. studied the efficacy of photobiomodulation and topical clonazepam therapy in BMS; there were indications that photobiomodulation is capable of reducing the symptoms in patients with BMS, with patients experiencing constant and long-lasting effects after the end of the first applications [29].

Melatonin (N-acetyl−5-methoxytryptamine) is best known for its participation in the regulation of circadian rhythms. In addition, melatonin has neuroprotective, anti-inflammatory, and immune-modulating actions [30,31,32,33,34,35]. On the other hand, melatonin is reported to have analgesic effects in application to neuropathic disorders, hyperalgesia, and allodynia [36]. The location and activity of its receptors have been associated with the main pain-regulating centers, including the trigeminal pathway and the trigeminal nucleus, which are intimately related to orofacial pain [36]. Likewise, melatonin binding to its receptors has been intimately related to the dopaminergic and GABAergic pathways. Thus, some studies have suggested that the application of melatonin acts in a way similar to certain benzodiazepines, such as clonazepam [30,34,36,37].

Therefore, we have hypothesized that melatonin-based treatment could be beneficial for people suffering from BMS. The present study evaluates the response to treatment with melatonin and clonazepam versus placebo in patients with BMS via assessment of clinical variables (such as burning sensation score, xerostomia, quality of life among others) and salivary biomarkers related to stress (salivary alpha-amylase, oxytocin) and the immune system (ADA, ferritin).

## 2. Material and Methods

A randomized prospective study was carried out on the efficacy of melatonin and clonazepam versus placebo in patients with BMS. Double-blinding was applied to the administration of melatonin and the placebo but not to clonazepam. The study was carried out at the Dental Clinic of Morales Meseguer University Hospital (University of Murcia, Spain). Randomization was performed by an external agent. All patients gave written informed consent to participate in the study, which involved a two-month treatment duration. The study protocol abided with the principles of the Declaration of Helsinki and was approved by the Bioethics Committee of the University of Murcia (reference: 2203/2018). The study is registered at ClinicalTrials.gov (accessed on 28 December 2018) with the identifier: NCT03788733. Melatonin and placebo randomization were conducted by a professional unrelated to the investigation and the randomization codes were kept in a sealed envelope until all the patients had completed the study.

We included 80 consecutive patients diagnosed with BMS according to the criteria of the Scala [2]. The patients were assigned in a 1:1:1 proportion to one of the treatments (melatonin, clonazepam, or placebo) using a computer-generated randomization algorithm. Individuals allergic to melatonin or clonazepam were excluded from the study, in the same way as patients working night shifts, pregnant or nursing women, subjects receiving estrogen or hormone replacement therapy, and individuals using vitamins or nutritional supplements in the month prior to participation in the study.

### 2.1. Study Protocol: Clinical Variables and Data Compilation

**Figure 1:**
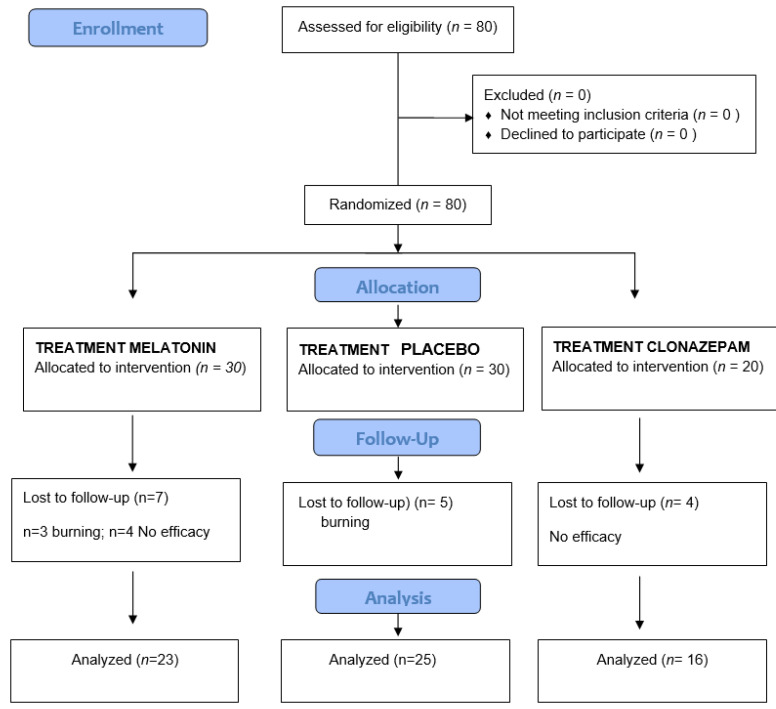
Flow Diagram.

The patients were divided into three groups: melatonin, clonazepam, and placebo. Following confirmation of the inclusion criteria, data compilation was carried out by a single investigator (CCF) (Figure 1). Patient age and gender were recorded, as well as smoking habits, alcohol intake, and oral hygiene. A buccodental exploration was carried out, with assessment of the pain symptoms and location of the burning sensation. Then, heart rate and arterial oxygen saturation (SatO2) were recorded by pulsioximetry. The following questionnaires were applied:

- **Burning sensation visual analog scale (VAS pain):** the patient scores burning sensation on a VAS from 0 to 10, where 0 = no burning sensation and 10 = maximum burning sensation [38].
- **Xerostomia score (VAS xeros)**: the patient scores xerostomia (dry mouth) on a VAS from 0 to 10, where 0 = no xerostomia and 10 = maximum xerostomia.
- **Oral Health Impact Profile 14 (OHIP-14)(Spanish version):** this questionnaire is composed of 7 dimensions: functional limitation, physical discomfort, psychological discomfort, physical disability, psychological disability, social disability, and handicaps. Each dimension comprises two questions (with a total of 14 questions), and the answers are scored from 0 to 4 (0 = lowest level and 4 = highest level). The higher the score, the poorer the oral quality of life of the patient [39].
- **Hospital Anxiety and Depression Scale (HADS):** this instrument consists of two subscales that respectively assess anxiety state (HADS-A) and depressive state (HADS-D). Each subscale has 7 items scored from 0 to 3, where a total score of over 10 reflects the presence of anxiety or depression, scores between 8 and 10 are borderline, and a score of under 7 indicates the absence of anxiety or depression [40].
- **Pittsburg Sleep Quality Index:** this instrument consists of 19 questions divided into 7 sections, with each section addressing a specific characteristic of the patient sleep pattern: subjective quality of sleep, latency of sleep, duration of sleep, usual efficiency of sleep, alterations of sleep, use of medication to sleep, and daytime dysfunction. Each section receives a score of 0–3, where 0 = no problems and 3 = great problems. The final score corresponds to the sum of the scores of the 7 sections, yielding a maximum score of 21 points. A score of 5 or less is indicative of satisfactory quality of sleep, while scores of over 5 are indicative of sleep disorders [41].
- **Epworth daytime sleepiness scale:** this scale comprises 8 questions that simulate situations in which the patient is asked to score the probability of experiencing sleepiness. The result of the questionnaire is the sum of the individual scores of the 8 questions, and higher scores are indicative of a greater probability of daytime sleepiness [42].

### 2.2. Sialometry

Resting saliva output was measured for 5 min, using the drainage technique. Measurements were always performed in the morning, taking into account the circadian rhythm, and following a fasting period of at least two hours. The saliva was collected in graded polyethylene tubes, which were visually inspected, and any samples containing blood were discarded. The samples were then immediately centrifuged (3000 rpm for 10 min at 4 °C), and the supernatant was stored at −80 °C until analysis.

With regard to both melatonin treatment and the administration of placebo, the patients received edible cellulose and starch strips allowing instant release and rapid absorption of the active ingredient (1 mg of melatonin per strip). Each strip was supplied in a sealed aluminum sachet for both melatonin and placebo (size 22 × 32 mm^2^, thickness < 1 mm). Neither the investigator nor the patient was aware of the treatment administered.

The clonazepam group received Rivotril^®^ 2.5 mg/mL oral solution, administering 5 drops (0.5 mg of clonazepam) in the morning and 5 drops at night in the form of a rinse retained in the mouth for 1.5 min [43,44]. The patients were instructed not to swallow the drug.

The duration of the treatments was two months, after which all the clinical variables were again recorded, together with patient satisfaction with the treatment and its organoleptic properties. We likewise recorded the possible presence of side effects during the two-month treatment period.

### 2.3. Biochemical Analysis

The salivary alpha-amylase activity was measured by a commercial kit using the International Federation of Clinical Chemistry and Laboratory Medicine method [18,20,23]. ADA was measured using a commercially available spectrophotometric assay (Adenosine Deaminase assay kit, Diazyme Laboratories, Poway, California, USA) adapted to an automated analyzer (Olympus AU400 automated biochemical analyzer, Olympus Diagnostica GmbH, Ennis, Ireland) [18]. Ferritin was measured by a commercial immunoturbidimetric assay that uses polyclonal anti-human ferritin antibodies (Tina-quant ferritin, Roche Diagnostics, Indianapolis, United States) in an automated analyzer (Olympus AU400 automated biochemical analyzer, Olympus Diagnostica GmbH, Ennis, Ireland) [23].

Salivary total protein content was determined using a colorimetric assay (protein in urine and CSF, Spinreact, Spain) adapted for its use in automatic analyzers (Olympus UA600 automated biochemical analyzer, Olympus Diagnostica GmbH, Ennis, Ireland) following the manufacturer’s instructions.

### 2.4. Statistical Analysis

Data validation was performed by assessing normal distribution (Shapiro–Wilk test), homoscedasticity (Levene’s test), and sphericity. In cases lacking any of these properties, and following log transformation, nonsignificant values were obtained in contrast testing, with all the data being considered valid for analysis. Statistical significance was considered for *p* < 0.05. The SPSS version 25.0 statistical package (SPSS Inc., New York, NY, USA) was used throughout.

## 3. Results

A total of 64 patients with a mean age of 57.8 years (range 39–83) were divided into three groups (melatonin, placebo, clonazepam). No statistically differences were detected between groups in terms of patient age, sex, oral hygiene, or smoking habits, with exception of alcohol consumption (Table 1).

The VAS pain for the intensity of the burning sensation as a dependent variable in the three treatment groups, before and after treatment, evidenced a decrease in the mean score in all three groups at the end of treatment. In the melatonin group, the mean (±standard deviation (SD)) score decreased from 7.8 ± 1.5 before treatment to 5.8 ± 2.54 after treatment (*p* < 0.001). In the clonazepam group, the score decreased from 8.8 ± 1.2 to 5.5 ± 3.6 (*p* < 0.01), and in the placebo group, the score decreased from 8.1 ± 1.8 to 7.7 ± 1.9, though in this case, the difference failed to reach statistical significance (*p* > 0.05).

With regard to the differences in decreased burning sensations between groups, significant differences were observed between the administration of melatonin versus placebo (*p* = 0.049), but not between clonazepam and the placebo (*p* = 0.590) or between the administration of clonazepam and melatonin (*p* = 0.290).

Likewise, with regard to xerostomia, a decrease was observed in the mean VAS xeros score in all three groups at the end of treatment, although without reaching statistical significance in any of them. In the melatonin group, the mean (± standard deviation [SD]) score decreased from 3.73 ± 3.7 before treatment to 3.26 ± 3.12 after treatment. In the clonazepam group, the score decreased from 4.68 ± 3.53 to 3.56 ± 2.80, and in the placebo group, the score decreased from 5.06 ± 3.63 to 4.93 ± 3.52.

The saliva drainage test was the dependent variable in all three treatment groups, and the results were evaluated before and after administration of each of the drugs. An increase was recorded in the melatonin group (from 3.47 ± 2.46 to 4.13 ± 2.56), with a slight decrease in the clonazepam group (from 2.65 ± 1.98 to 2.47 ± 1.69), and no changes in the placebo group (2.85 ± 1.63 before treatment versus 2.85 ± 2.08 after treatment). The difference proved statistically significant in the melatonin group (*p* = 0.04). No significant differences were observed in the comparison of the changes in salivary drainage between the groups (*p* > 0.05).

The Oral Health Impact Profile 14 (OHIP-14) corresponding to perceived oral quality of life evidenced a decrease in the mean score in all three study groups. The largest decrease was recorded in the clonazepam group (36 ± 10.00 before treatment versus 28.13 ± 8.82 after treatment), followed by the melatonin group (31 ± 7.80 before treatment versus 27.7 ± 8.11 after treatment) and the placebo group (35.5 ± 10.95 before treatment versus 34.48 ± 11.46 after treatment). The decrease proved significant for melatonin (*p* < 0.001) and clonazepam (*p* = 0.001), but not for the placebo (*p* = 0.133). In turn, significant differences were recorded between melatonin and the placebo (*p* = 0.036), but not in the rest of the comparisons between groups.

The anxiety levels were measured with the Hospital Anxiety and Depression Scale (HADS). Regarding the items referred to anxiety, the highest scores corresponded to the clonazepam group, which showed the greatest reductions of anxiety (11.25 ± 4.59 before treatment versus 9.81 ± 4.32 after treatment), followed by melatonin (9.13 ± 3.84 versus 8.78 ± 4.17), while the placebo group showed a slight increase (10.24 ± 4.48 versus 10.40 ± 4.32). Despite the variations in mean scores, there were no significant differences between the pre- and post-treatment scores in any of the groups, or in the comparisons between groups (*p* > 0.05 in all cases).

With regard to depression, the melatonin group showed a slight increase in score (4.17 ± 3.12 before treatment versus 4.22 ± 3.33 after treatment), while the clonazepam group showed a slight decrease (6.25 ± 4.48 versus 5.81 ± 4.43). A slight increase in the depression score was recorded in the placebo group (4.60 ± 5.33 before treatment versus 4.76 ± 5.31 after treatment). There were no significant differences between the pre- and post-treatment scores in any of the groups, or in the comparisons between groups (*p* > 0.05 in all cases).

The quality of sleep was assessed using the Pittsburg Sleep Quality Index. There were no significant differences between the first and second visits in any group, though the clonazepam group showed values very close to statistical significance (*p* = 0.055). In turn, the Epworth Daytime Sleepiness Scale showed no significant differences between the pre- and post-treatment sleepiness scores in any of the groups, or in the comparisons between groups.

The evaluation of the number of locations of burning mouth sensation before and after each of the treatments showed a decrease in the number of locations in all three groups: melatonin 3.13 ± 1.45 before treatment versus 2.61 ± 1.46 after treatment; clonazepam 3.60 ± 1.35 versus 2.94 ± 1.84; and placebo 3.92 ± 1.28 versus 3.60 ± 1.44. The difference was shown to be statistically significant in the melatonin group (*p* = 0.015). In the comparison between treatment groups, significant differences were observed between melatonin and placebo (*p* = 0.028).

Eight patients in the global sample (12.5%) presented adverse effects, all of which were mild: five in the melatonin group and three in the placebo group, with no adverse effects in the clonazepam group. The recorded adverse effects corresponded to mild gastrointestinal alterations, and none of them prevented the patients from completing the two months of the study.

No changes in saliva composition were observed in relation to oxytocin, ferritin, ADA, amylase, or uric acid before versus after the administration of treatment, though significantly higher total protein content was recorded in the melatonin group after the treatment versus pre-treatment (Table 2).

On evaluating the pre- versus post-treatment changes in each clinical variable with respect to the variations in salivary composition, correlations were observed between oxytocin and saliva drainage (r = −0.410; *p* = 0.009), heart rate and salivary amylase (r = 0.346; *p* = 0.029), oxygen saturation and salivary amylase (r = 0.419; *p* = 0.007), and the HAD-D score and ferritin (r = −0.312; *p* = 0.05), and ADA and protein total (r = 0.56; *p* = 0.001).

## 4. Discussion

In the present study, melatonin and clonazepam were shown to be effective at reducing burning mouth sensation and in improving the patient’s quality of life compared with the placebo. Both melatonin and clonazepam were safe, with no major adverse effects recorded in the patients with BMS.

Melatonin has been reported to exert beneficial effects upon its target organs, with anti-inflammatory actions and a reduction of oxidative stress that protects against cell damage [31,32,45]. Some studies have found melatonin to be able to neutralize free radicals and protect the mitochondria against oxidative stress, with the restoration of normal mitochondrial functioning in different inflammatory disease conditions [30,34]. Due to this range of activities, melatonin has been attributed with neuroprotective, antitumor, and tissue regeneration properties [30,31,32,33,34,35,36].

The only study to date on the application of melatonin in BMS was published by Varoni et al. [37]. The authors reported no improvement in the intensity of burning sensation and no significant differences versus the placebo, despite the administration of higher doses than in our own study (12 mg/day). This suggests that higher doses do not necessarily imply improved therapeutic outcomes—though the risk of adverse effects may increase. In our study, all three treatments (melatonin, clonazepam, and placebo) resulted in a decrease in the number of sites of burning sensation, though significant differences were only observed in the melatonin group. In this regard, administration in the form of soft bands might be a method worth considering.

With regard to saliva drainage, melatonin was associated with a significant increase in salivary levels following treatment (*p* = 0.004), coinciding with the observations of other authors [34]. Melatonin may be useful as a coadjuvant in the treatment of certain disorders of the oral cavity, improving salivation [34,36]. Since dry mouth is present in many patients with BMS, melatonin could offer marked improvement of quality of life.

The scientific evidence on the appropriate melatonin dose for the management of sleep disturbances is limited, with figures ranging between 0.5 and 10 mg [46]. In the present study, we did not specifically address improvements in the quality of sleep, and the absence of results of clinical relevance may have been due to the administration of an insufficient drug dose.

Both anxiety and depression were shown to decrease in the two active drug treatment groups, while the placebo group exhibited a slight increase in score. The greatest reduction of anxiety corresponded to clonazepam, though statistical significance was not reached. In contrast, Varoni et al. [37] documented a significant decrease in anxiety with melatonin versus a placebo. The differences in results obtained may possibly be explained by the lower drug dosage used in our study.

The literature offers heterogeneous data regarding the application of clonazepam in patients with BMS [8,26]. It is one of the drugs of choice for the treatment of sleep disturbances, although the studies in which clonazepam is used in application to BMS focus mainly on the reduction of the burning sensation, without addressing the quality of sleep [43]. The application of clonazepam can afford a rapid analgesic effect that may prove effective at reducing the burning sensation in BMS through stabilization of the nerve fiber membrane and cells of the oral mucosa [44,45,46]. Cui et al. [27], in a review of the application of clonazepam in BMS, were unable to establish optimum topical or systemic doses in patients with this syndrome; in this regard, the reported duration, method of administration and doses of clonazepam used to treat BMS varied. Previous studies describe dryness, drowsiness, and fatigue as the most common side effects of clonazepam, and the drug, moreover, may cause dependency [27,44,45,46]. Adverse effects of melatonin are not serious and are infrequent; headache and somnolence, nausea, palpitations, and abdominal pain [47] Circadian clock dysfunction is an emerging area of research that may underpin many BMS disease manifestations. Pain perception, depression and anxiety, and sleep disorders are inextricably linked with circadian disturbances. Melatonin may regulate and restore circadian rhythms [6,30]. Therefore, more investigations into the mechanism of melatonin in burning mouth syndrome are necessary.

It is important to mention the placebo effect of treatments in BMS. Kuten-Shorrer et al. [48] published a meta-analysis in which a large percentage of patients (up to 72%) responded favorably to the application of a placebo. The authors thus suggested that the type of placebo used in clinical studies on BMS should be standardized, with the conduction of at least 8 weeks of follow-up, and the inclusion of “no treatment” groups, in order to ensure greater reliability in evaluating the effectiveness of the studied treatments [48].

Saliva is one of the defense systems of the body and could prove useful as a diagnostic fluid. Burning mouth syndrome is a challenge for clinicians; current research therefore should focus on methods combining physiological parameters, psychological and behavioral aspects, and the characteristics of saliva [12,18,19,20,21,22,23,24,49]. We observed a negative correlation to drainage and oxytocin when analyzing the changes in the composition of saliva in relation to the post-treatment clinical parameters. The latter is a neuropeptide found in the nervous system and has been implicated in pain attenuation. Variations in the endogenous oxytocin system are associated to the morphology of the brain regions implicated in pain processing and modulation. Oxytocin induces analgesia, and different studies have shown that the production of this neuropeptide contributes to cope with situations of stress and anxiety, and strengthens affectivity [19,22]. This is crucial in treating problems of anxiety and stress. In our study, oxytocin in saliva was seen to increase in all the groups after treatment, with no significant differences in any of the groups.

In recent years, salivary alpha-amylase (AA) has been shown to be a valid and reliable indicator of the activity of the autonomic nervous system (ANS) in situations of stress. It is therefore a biomarker worth considering [18,19,49,50,51]. High salivary alpha-amylase levels reflect adrenergic activity secondary to biological–psychosocial stress of the adrenal gland medullary sympathetic system; salivary AA is therefore considered to be a biological marker of psychological stress. These systems closely interact with the immune system and are thus implicated in the development and maintenance of pathological conditions. Nagler et al. [50] indicated that patients with BMS are chronically exposed to stress because of the persistent oral pain. In our study, we observed a relationship between salivary AA and certain clinical parameters, such as heart rate (*p* = 0.029) and oxygen saturation (*p* = 0.007).

The measurement of adenosine deaminase (ADA) can yield information on cell mediated immunity. This enzyme hydrolyses adenosine and is widely distributed in the different body tissues. It has been shown to play a role in the function, maturation, and maintenance of immune responses. In our study, no association was observed between ADA and the different clinical parameters evaluated. The increase in adenosine levels during the course of the day contributes to drowsiness [52]. Due to the close relationship between mood state and sleep behavior, further research in this field may reveal data on the progression of different psychological disorders, such as chronic anxiety and stress.

Ferritin is an iron storing protein that plays a key role in cellular oxygen metabolism, and its measurement has been shown to be useful for assessing intracellular iron status [18,23]. Moreover, in recent years, ferritin has been described as a key immune system molecule, with immunosuppressive properties and proinflammatory effects, and a number of studies have demonstrated its role as an acute phase reactant. In our study, ferritin was shown to be correlated to depression—an observation that requires more in-depth investigation.

## 5. Study Limitations

In the present study, double-blinding was applied to the administration of melatonin and a placebo but not to clonazepam, which was administered in the form of drops (oral solution), since it could not be administered in the form of strips, such as melatonin or a placebo. Despite the noninvasive nature of salivary analysis, this body fluid has not yet become popular in research. More in-depth research is therefore required of the different salivary components, due to the broad interindividual ranges involved, with the recruitment of larger cohorts and longer term assessments of patient responses to treatment.

## 6. Conclusions

Melatonin and clonazepam were shown to be effective at reducing the burning sensation and improving quality of life. Both drugs were found to be safe, with no major adverse effects in patients with BMS. Melatonin may be regarded as an alternative treatment for VAS pain patients with BMS, although further studies are needed to confirm its effectiveness and define the optimum dosage.

## Figures and Tables

**Table 1:** Description and comparison of the demographic data and habits of the study subjects.

	Melatonin	Clonazepam	Placebo	*p*
***Gender (%)*** *Female* *Male*	19 (82.61) 4 (17.39)	15 (92) 1 (6.25)	23 (92) 2 (8)	*p* > 0.05
***Age (mean;SD)***	57.68 (10.32)	63.81 (10.69)	60.40 (13.8)	*p* > 0.05
***Smoking n (%)*** *Smoking* *No Smoking* *Ex Smoking*	6 (26.1) 13 (56.5) 4 (17.4)	3 (18.8) 13 (81.3) 0 (0)	4 (16) 17 (68) 4 (16)	*p* > 0.05
***Alcohol n (%)*** *less than once a week* *Once a day* *weekends*	11 (47.8) 6 (26.1) 6 (26.1)	15 (93.7) 0 (0) 1 (6.3)	20 (80) 2 (8) 3 (12)	*p* < 0.05
***Oral hygiene (brushing) n (%)*** *No* *Once a day* *Twice a day* *Three times a day*	0 (0) 13 (59.9) 0(0) 9 (39.1)	1 (6.3) 7 (43.8) 1 (6.3) 7 (43.8)	1(4) 10 (40) 1 (4) 13 (52)	*p* > 0.05

**Table 2:** Pre vs Post different treatments.

	Melatonin		*p*	Placebo		*p*	Clonazepam		*p*
	Pre	Post		Pre	Post		Pre	Post	
Oxytocin	804.4 (562–1902)	1581 (544.6–2128)	0.339	1417 (907.6–2491)	1627 (768.4–2388)	0.953	804.4 (562–3065)	1581 (544.6–2467)	0.910
ADA	3.1 (0.75–5.7)	2.8 (0.75–6.575)	0.718	3.8 (1.3–11.1)	3.7 (1.4–11.5)	0.992	3.1 (0.75–7.525)	2.8 (0.75–5.65)	0.945
Ferritin	6$(2–13.78)	5.75 (3.075–22.18)	0.266	9 (4.25–12.33)	8.45 (2.925–19.7)	0.252	6 (2–22.63)	5.75 (3.075–17.55)	0.219
SAA (U/L)	198,760 (132,680–368,600)	196,720 (111,080–359,160)	0.266	181,520 (97,400–364,520)	166,960 (81,680–232,920)	0.891	198,760 (132,680–395,180)	196,720 (111,080–382,800)	0.910
PT	121.9 (78.35–208.4)	149.6 (101.2–222.1)	0.043	185.9 (99.38–309.1)	130 (90.16–332.2)	0.679	121.9 (78.35–183.2)	149.6 (101.2–217.5)	0.250

Note: ADA = adenosine deaminase; SAA, salivary alpha-amylase; Pt Total proteins.

## Data Availability

Data is contained within the article.

## References

[1] Jääskeläinen S.K., Woda A. (2017). Burning mouth syndrome. Cephalalgia.

[2] Scala A., Checchi L., Montevecchi M., Marini I., Giamberardino M.A. (2003). Update on Burning Mouth Syndrome: Overview and Patient Management. Crit. Rev. Oral Biol. Med..

[3] Kohorst J.J., Bruce A.J., Torgerson R.R., Schenck L.A., Davis M.D.P. (2015). The prevalence of burning mouth syndrome: A population-based study. Br. J. Dermatol..

[4] Orofacial Pain Classification Committee (2020). International Classification of Orofacial Pain, 1st edition (ICOP). Cephalalgia.

[5] Forssell H., Teerijoki-Oksa T., Kotiranta U., Kantola R., Bäck M., Vuorjoki-Ranta T.-R., Siponen M., Leino A., Puukka P., Estlander A.-M. (2012). Pain and pain behavior in burning mouth syndrome: A pain diary study. J. Orofac. Pain.

[6] Schiavone V., Adamo D., Ventrella G., Morlino M., De Notaris E.B., Ravel M.G., Kusmann F., Piantadosi M., Pollio A., Fortuna G. (2012). Anxiety, Depression, and Pain in Burning Mouth Syndrome: First Chicken or Egg?. Headache.

[7] Silvestre F.J., Silvestre-Rangil J., López-Jornet P. (2015). Burning mouth syndrome: A review and update. Rev. Neurol..

[8] Tan H.L., Smith J.G., Hoffmann J., Renton T. (2022). A systematic review of treatment for patients with burning mouth syndrome. Cephalalgia.

[9] Klasser G.D., Fischer D.J., Epstein J.B. (2008). Burning Mouth Syndrome: Recognition, Understanding, and Management. Oral Maxillofac. Surg. Clin. N. Am..

[10] López-Jornet P., Camacho-Alonso F., Andujar-Mateos P., Sanchez-Siles M., Gomez-Garcia F. (2010). Burning mouth syndrome: An update. Med. Oral Patol. Oral Cir. Bucal.

[11] Ariyawardana A., Chmieliauskaite M., Farag A.M., Albuquerque R., Forssell H., Nasri-Heir C., Klasser G.D., Sardella A., Mignogna M.D., Ingram M. (2019). World Workshop on Oral Medicine VII: Burning mouth syndrome: A systematic review of disease definitions and diagnostic criteria utilized in randomized clinical trials. Oral Dis..

[12] Lopez-Jornet P., Felipe C.C., Pardo-Marin L., Ceron J.J., Pons-Fuster E., Tvarijonaviciute A. (2020). Salivary Biomarkers and Their Correlation with Pain and Stress in Patients with Burning Mouth Syndrome. J. Clin. Med..

[13] Kim H.-I., Kim Y.-Y., Chang J.-Y., Ko J.-Y., Kho H.-S. (2012). Salivary cortisol, 17β-estradiol, progesterone, dehydroepiandrosterone, and α-amylase in patients with burning mouth syndrome. Oral Dis..

[14] Carreño-Hernández I., Cassol-Spanemberg J., Rodríguez de Rivera-Campillo E., Estrugo-Devesa A., López-López J. (2021). Is Burning Mouth Syndrome a Neuropathic Pain Disorder? A Systematic Review. J. Oral Facial Pain Headache.

[15] Lee G.S., Kim H.K., Kim M.E. (2022). Relevance of sleep, pain cognition, and psychological distress with regard to pain in patients with burning mouth syndrome. Cranio.

[16] Ritchie A., Kramer J. (2018). Recent Advances in the Etiology and Treatment of Burning Mouth Syndrome. J. Dent. Res..

[17] Lee Y.C., Hong I.K., Na S.Y., Eun Y.G. (2015). Evaluation of salivary function in patients with burning mouth syndrome. Oral Dis..

[18] Franco-Martínez L., Tecles F., Torres-Cantero A., Bernal E., San Lázaro I., Alcaraz M.J., Vicente-Romero M.R., Lamy E., Sánchez-Resalt C., Rubio C.P. (2021). Analytical validation of an automated assay for the measurement of adenosine deaminase (ADA) and its isoenzymes in saliva and a pilot evaluation of their changes in patients with SARS-CoV-2 infection. Clin. Chem. Lab. Med..

[19] Ngamchuea K., Chaisiwamongkhol K., Batchelor-Mcauley C., Compton R.G. (2018). Chemical analysis in saliva and the search for salivary biomarkers—A tutorial review. Analyst.

[20] Tvarijonaviciute A., Zamora C., Martinez-Subiela S., Tecles F., Pina F., Lopez-Jornet P. (2019). Salivary adiponectin, but not adenosine deaminase, correlates with clinical signs in women with Sjögren’s syndrome: A pilot study. Clin. Oral Investig..

[21] Orbach H., Zandman-Goddard G., Amital H., Barak V., Szekanecz Z., Szucs G., Danko K., Nagy E., Csepany T., Carvalho J.F. (2007). Novel Biomarkers in Autoimmune Diseases: Prolactin, Ferritin, Vitamin D, and TPA Levels in Autoimmune Diseases. Ann. N. Y. Acad. Sci..

[22] Mera J.C., Molano M.A., López C.C., Triana C.A., Cotrina J.M. (2021). Discussions and perspectives regarding oxytocin as a biomarker in human investigations. Heliyon.

[23] Franco-Martínez L., Tvarijonaviciute A., Martínez-Subiela S., Márquez G., Martínez Díaz N., Cugat R., Cerón J.J., Jiménez-Reyes P. (2019). Changes in lactate, ferritin, and uric acid in saliva after repeated explosive effort sequences. J. Sports Med. Phys. Fit..

[24] Nater U.M., Rohleder N. (2009). Salivary alpha-amylase as a non-invasive biomarker for the sympathetic nervous system: Current state of research. Psychoneuroendocrinology.

[25] Lokesh Sundaram B., Rathnavelu V., Sabesan M., Ganesh A., Anandan S. (2021). A Study to Assess the Levels of Salivary Ferritin in Iron Deficiency Anemia Subjects and Healthy Subjects. Cureus.

[26] McMillan R., Forssell H., Buchanan J.A., Glenny A.M., Weldon J.C., Zakrzewska J.M. (2016). Interventions for treating burning mouth syndrome. Cochrane Database Syst. Rev..

[27] Cui Y., Xu H., Chen F.M., Liu J.L., Jiang L., Zhou Y., Chen Q.M. (2016). Efficacy evaluation of clonazepam for symptom remission in burning mouth syndrome: A meta-analysis. Oral Dis..

[28] Liu Y.F., Kim Y., Yoo T., Han P., Inman J.C. (2018). Burning mouth syndrome: A systematic review of treatments. Oral Dis..

[29] Arduino P.G., Cafaro A., Garrone M., Gambino A., Cabras M., Romagnoli E., Broccoletti R. (2016). A randomized pilot study to assess the safety and the value of low-level laser therapy versus clonazepam in patients with burning mouth syndrome. Lasers Med. Sci..

[30] Varoni E.M., Soru C., Pluchino R., Intra C., Iriti M. (2016). The Impact of Melatonin in Research. Molecules.

[31] Abdel Moneim A.E., Guerra-Librero A., Florido J., Shen Y.Q., Fernández-Gil B., Acuña-Castroviejo D., Escames G. (2017). Oral Mucositis: Melatonin Gel an Effective New Treatment. Int. J. Mol. Sci..

[32] Carpentieri A.R., Peralta Lopez M.E., Aguilar J., Solá V.M. (2017). Melatonin and periodontal tissues: Molecular and clinical perspectives. Pharmacol. Res..

[33] Elsabagh H.H., Moussa E., Mahmoud S.A., Elsaka R.O., Abdelrahman H. (2020). Efficacy of Melatonin in prevention of radiation-induced oral mucositis: A randomized clinical trial. Oral Dis..

[34] Reiter R.J., Rosales-Corral S.A., Liu X.Y., Acuña-Castroviejo D., Escames G., Tan D.-X. (2015). Melatonin in the oral cavity: Physiological and pathological implications. J. Periodontal Res..

[35] Permuy M., López-Peña M., González-Cantalapiedra A., Muñoz F. (2017). Melatonin: A Review of Its Potential Functions and Effects on Dental Diseases. Int. J. Mol. Sci..

[36] Kuthati Y., Lin S., Chen I., Wong C. (2018). Melatonin and their analogs as a potential use in the management of Neuropathic pain. J. Formos. Med Assoc..

[37] Varoni E.M., Lo Faro A.F., Lodi G., Carrassi A., Iriti M., Sardella A. (2018). Melatonin Treatment in Patients with Burning Mouth Syndrome: A Triple-Blind, Placebo-Controlled, Crossover Randomized Clinical Trial. J. Oral Facial Pain Headache.

[38] Carlsson A.M. (1983). Assessment of chronic pain. I. Aspects of the reliability and validity of the visual analogue scale. Pain.

[39] Montero-Martín J., Bravo-Pérez M., Albaladejo-Martínez A., Hernández-Martín L.A. (2009). Validation the Oral Health Impact Profile (OHIP-14sp) for adults in Spain. Med. Oral Patol. Oral Cir. Bucal.

[40] Zigmond A.S., Snaith R.P. (1983). The hospital anxiety and depression scale. Acta Psychiatr. Scand..

[41] Buysse D.J., Reynolds C.F., Monk T.H., Berman S.R., Kupfer D.J. (1989). The Pittsburgh Sleep Quality Index: A new instrument for psychiatric practice and research. Psychiatry Res..

[42] Johns M.W. (1991). A New Method for Measuring Daytime Sleepiness: The Epworth Sleepiness Scale. Sleep.

[43] Patton L.L., Siegel M.A., Benoliel R., De Laat A. (2007). Management of burning mouth syndrome: Systematic review and management recommendations. Oral Surg. Oral Med. Oral Pathol. Oral Radiol. Endodontology.

[44] Kuten-Shorrer M., Treister N.S., Stock S., Kelley J.M., Ji Y.D., Woo S.B., Lerman M.A., Palmason S., Sonis S.T., Villa A. (2017). Topical Clonazepam Solution for the Management of Burning Mouth Syndrome: A Retrospective Study. J. Oral Facial Pain Headache.

[45] Vecchierini M.F., Kilic-Huck U., Quera-Salva M.A. (2021). Melatonin (MEL) and its use in neurological diseases and insomnia: Rec-ommendations of the French Medical and Research Sleep Society (SFRMS). Rev. Neurol..

[46] Rossella I., Alessandro V., Naman R., Gary K., Hervé S.Y. (2022). Topical clonazepam for burning mouth syndrome: Is it efficacious in patients with anxiety or depression?. J. Oral Rehabil..

[47] Kuten-Shorrer M., Kelley J.M., Sonis S.T., Treister N.S. (2014). Placebo effect in burning mouth syndrome: A systematic review. Oral Dis..

[48] Costello R.B., Lentino C.V., Boyd C.C., O’connell M.L., Crawford C.C., Sprengel M.L., Deuster P.A. (2014). The effectiveness of melatonin for promoting healthy sleep: A rapid evidence assessment of the literature. Nutr. J..

[49] Melguizo-Rodríguez L., Costela-Ruiz V.J., Manzano-Moreno F.J., Ruiz C., Illescas-Montes R. (2020). Salivary Biomarkers and Their Application in the Diagnosis and Monitoring of the Most Common Oral Pathologies. Int. J. Mol. Sci..

[50] Nagler R.M., Hershkovich O. (2004). Sialochemical and gustatory analysis in patients with oral sensory complaints. J. Pain.

[51] Imura H., Shimada M., Yamazaki Y., Sugimoto K. (2016). Characteristic changes of saliva and taste in burning mouth syndrome pa-tients. J. Oral Pathol. Med..

[52] Tartar J.L., Hiffernan F.S., Freitas K.E., Fins A.I., Banks J.B. (2021). A Functional Adenosine Deaminase Polymorphism Associates with Evening Melatonin Levels and Sleep Quality. J. Circadian Rhythm..

